# Integrated Application of Thiourea and Biochar Improves Maize Growth, Antioxidant Activity and Reduces Cadmium Bioavailability in Cadmium-Contaminated Soil

**DOI:** 10.3389/fpls.2021.809322

**Published:** 2022-01-28

**Authors:** Fasih Ullah Haider, Ahmad Latif Virk, Muhammad Ishaq Asif Rehmani, Milan Skalicky, Syed Tahir Ata-ul-Karim, Naeem Ahmad, Walid Soufan, Marian Brestic, Ayman E. L. Sabagh, Cai Liqun

**Affiliations:** ^1^College of Resources and Environmental Sciences, Gansu Agricultural University, Lanzhou, China; ^2^College of Agronomy and Biotechnology, China Agricultural University, Key Laboratory of Farming System, Ministry of Agriculture and Rural Affairs of China, Beijing, China; ^3^Departmet of Agronomy, Ghazi University, Dera Ghazi Khan, Pakistan; ^4^Department of Botany and Plant Physiology, Faculty of Agrobiology, Food and Natural Resources, Czech University of Life Sciences Prague, Prague, Czechia; ^5^Graduate School of Agricultural and Life Sciences, The University of Tokyo, Tokyo, Japan; ^6^College of Agronomy, Northwest A&F University, Yangling, China; ^7^Department of Plant Production, College of Food and Agriculture, King Saud University, Riyadh, Saudi Arabia; ^8^Institute of Plant and Environmental Sciences, Faculty of Agrobiology and Food Resources, Slovak University of Agriculture, Nitra, Slovakia; ^9^Department of Agronomy, Faculty of Agriculture, Kafrelsheikh University, Kafr el-Sheikh, Egypt

**Keywords:** biochar, cadmium toxicity, thiourea, maize growth, antioxidant

## Abstract

Cadmium (Cd) contamination of croplands jeopardizes sustainable crop production and human health. However, curtailing Cd transfer and mobility in the rhizosphere-plant system is challenging. Sole application of biochar (BC) and thiourea (TU) has been reported to restrain Cd toxicity and uptake in plants. However, the combined applications of BC and TU in mitigating the harmful effects of Cd on plants have not yet been thoroughly investigated. Therefore, this study attempts to explore the integrated impact of three maize stalk BC application rates [*B*_0_ (0% w/w), *B*_1_ (2.5% w/w), and *B*_2_ (5% w/w)] and three TU foliar application rates [*T*_0_ (0 mg L^–1^), *T*_1_ (600 mg L^–1^), and *T*_2_ (1,200 mg L^–1^)] in remediating the adverse effects of Cd on maize growth, development, and physiology. Results demonstrated that Cd concentration in soil inhibited plant growth by reducing leaf area, photosynthesis activity, and enhanced oxidative stress in maize. Nevertheless, BC and TU application in combination (*B*_2_*T*_2_) improved the fresh biomass, shoot height, leaf area, and photosynthesis rate of maize plants by 27, 42, 36, and 15%, respectively, compared with control (*B*_0_*T*_0_). Additionally, the oxidative stress values [malondialdehyde (MDA), hydrogen peroxide (H_2_O_2_), and electrolyte leakage (EL)] were minimized by 26, 20, and 21%, respectively, under *B*_2_*T*_2_ as compared with *B*_0_*T*_0_. Antioxidant enzyme activities [superoxide dismutase (SOD) and catalase (CAT)] were 81 and 58%, respectively, higher in *B*_2_*T*_2_ than in *B*_0_*T*_0_. Besides, the shoot and root Cd concentrations were decreased by 42 and 49%, respectively, under *B*_2_*T*_2_ compared with *B*_0_*T*_0_. The recent study showed that the integrated effects of BC and TU have significant potential to improve the growth of maize on Cd-contaminated soil by reducing Cd content in plant organs (shoots and roots).

## Graphical Abstract

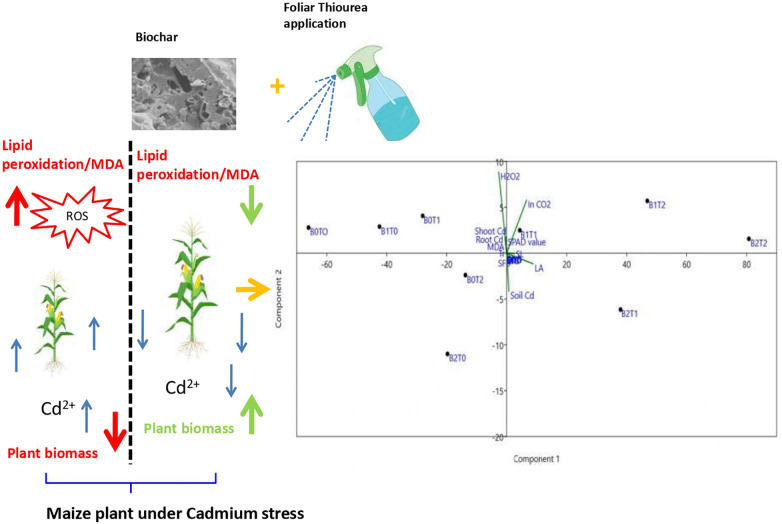

## Highlights

- Biochar (BC) application reduces the Cd uptake in maize plants.
- Thiourea (TU) application improves the plant growth and reduces the Cd uptake.
- The combined application of BC and TU could be profitable for Cd contamination.

## Introduction

Cadmium (Cd) is a very toxic trace element found in soil. It has been identified as a carcinogenic element and poses severe threats to human health (Haider et al., 2021). The primary sources of Cd contamination of croplands are anthropogenic activities, including fertilization, mining, and sewage sludge application which require additional study to alleviate Cd pollution and assure food security for the ever-growing global population (Wu et al., 2015). Cd availability and translocation in the soil-plant system substantially affect soil ecosystem services, reducing crop growth, yield, and quality (Zhao, 2020). Accumulation of Cd in the grains and other edible parts is the primary source of human exposure to contaminated food. Therefore, reducing health risk, particularly for crops cultivated on Cd contaminated soils, has been recently focused on (Zhang et al., 2009). Cd enters into human food chains and creates complicated health problems after being readily transported from Cd-contaminated soils to plant tissues (Ahmad et al., 2016). Plants grown on Cd-contaminated soil are subjected to osmotic stress, causing physiological degradation due to a reduction in relative leaf water content, transpiration, stomatal conductance, and photosynthetic activity (Anjum et al., 2017). Cd accumulation in plants not only damages chloroplast tissues, membrane permeability, and cell necrosis (Rafique et al., 2019) but also enhances the formation of reactive oxygen species (ROS). Therefore, an appropriate approach to remedy the harmful effects of Cd contamination is to limit the bioavailability of Cd in the rhizosphere to reduce its uptake by plants (Wang et al., 2019). Reducing Cd uptake by plants is also imperative for ensuring food safety. The current focus of the study has been on the decrease of Cd availability to crop plants.

Due to its higher ability to accumulate high Cd concentration, maize is ranked first among cereal crops, earning Cd in most plant organs as one of the leading crops. Besides, maize is one of the significant cereal crops in China, and most maize planting regions in China are Cd contaminated (Kollárová et al., 2019). Minimizing the bioavailability of Cd in the rhizosphere and reducing the uptake and accumulation of Cd by plants could effectively reduce the risk to human health and increase crop growth and yield (Zhu et al., 2020). Biochar (BC) has attained significant interest as a soil amendment for increasing carbon sink (Virk et al., 2020), mitigating greenhouse gas emission, and minimizing the Cd bioavailability in the rhizosphere (Younis et al., 2016). In addition, the role of BC in improving plant water availability, soil base saturation, and corn yield and minimizing Cd bioavailability in the rhizosphere has been widely reported (AbdElgawad et al., 2020). This decrease in Cd bioavailability might be attributed to the high absorption capacity and higher surface area of applied BC. The physicochemical properties of BC, such as surface area, surface charge, and pore spaces, and BC production parameters, such as pyrolysis retention time, pyrolysis temperature, and feedstock type, are major variables (Younis et al., 2016) that determine the adsorption capacity of Cd in contaminated agricultural soils (Khanna et al., 2019a).

The exogenous application of growth regulators has the potential to enhance plant growth and reduce the plant capacity to accumulate heavy metals. Thiourea (TU), a plant growth hormone, is actively involved in a series of developmental, physiological (Perveen et al., 2015), and biochemical changes in plants. Exogenous application of TU can mitigate Cd toxicity. Seed priming or foliar application of TU can play an imperative role in Cd detoxification and minimize Cd levels in plant tissues (Sharma and Dietz, 2009). Sulfur, the constituent of TU, improves plant metabolism, biochemical reactions (Srivastava et al., 2017), and protein folding and curtail plant Cd uptake.

Moreover, sulfur is also an essential constituent of tripeptide glutathione (GSH), which plays a critical function in plant redox regulation found on TU. Application of TU under Cd toxicity was found to improve leaf area expansion, chlorophyll *b*, stomatal conductance, photosynthesis rate, carotenoids in maize, and Cd transport from the rhizosphere to plant tissues. Moreover, exogenous application of TU has also been documented to decrease the uptake of arsenic in rice (Younis et al., 2016; Srivastava et al., 2017).

Although, the application of BC or TU alone has been reported to mitigate trace-metal bioavailability and enhance maize productivity. However, to the best of our understanding, the simultaneous application of BC application and TU to reduce Cd bioavailability and plant Cd uptake has not been studied yet. To this end, this study hypothesizes that the integrated application of BC and TU could reduce Cd uptake by reducing Cd bioavailability in the rhizosphere and maintaining antioxidant activity in plants than BC/TU application alone. Thus, this experiment was intended to investigate the impacts of combined application of BC and TU on mitigating Cd uptake in maize by evaluating the shoot and root growth, plant metabolic, and redox balance responses in maize grown on Cd contaminated soil.

## Materials and Methods

### Experiment Design and Management

A pot trial was performed using a complete randomized design (CRD) having factorial arrangement having three replications in the wirehouse of College of Resources and Environmental Sciences, Gansu Agricultural University, Lanzhou, China in the first week of July 2020 to mid-week of September 2020. The wirehouse was wrapped with a polythene sheet to protect the plants from rainwater. Metrological data, i.e., temperature, humidity, rainfall, and sunshine duration, were mentioned in Supplementary Table 1. Maize straw-derived BC was applied at three rates, namely (*B*_0_) 0% w/w, (*B*_1_) 2.5% w/w, and (*B*_2_) 5.0% w/w, mixed in the soil before pot filling. The BC used in the current experiment was manufactured from maize (*Zea may* L) straw at a pyrolysis temperature of 550°C (Zhou et al., 2019). The chemical and physical properties of BC and soil are presented in Table 1. Additionally, three TU dose rates were applied, namely, (*T*_0_) 0 mg L^–1^, (*T*_1_) 600 mg L^–1^, and (*T*_2_) 1,200 mg L^–1^ at 20 and 40 days after sowing (DAS). Before BC addition, well-mixed air-dried soil was spiked artificially with 30 ppm Cd using Cd nitrate as a source of Cd. Each containing 5 kg soil, experimental pots were randomly placed during the experiment to minimize location effects (Haider et al., 2021). Before sowing, maize cultivar Liyu-16 (LY-16) seeds were sterilized with 10% H_2_O_2_ solution (v/v) for 15 min and then washed with distilled water. The seeds of maize variety (LY-16) were acquired from Hebei Maohua Seed Industry Co., Ltd., Maohua, China. Two grains of maize were sown in each pot, and the latter one was uprooted to maintain one plant in each pot. Every pot was fertilized properly with 150 g of N, 100 g of P_2_O_5_, and 50 g of K_2_O, using urea (CH4N2O), diammonium phosphate [(NH_4_)_2_HPO_4_], and sulfate of potash (K_2_SO_4_), respectively. The optimum amount of water was applied to maintain the requirements of the growing plant.

**TABLE 1:** Physiochemical properties of biochar and soil used in the experiment.

Parameters	Maize straw biochar	Soil
pH	8.60	8.64
EC (mS cm^–1^)	3.78	2.94
Total N (g kg^–1^)	0.14	0.37
Total K %	17.30	–
Total P %	0.19	0.77
Total C (g kg^–1^)	6.70	3.71
Organic matter %	–	6.40
Available P mg kg^–1^	–	161.08
Available K mg kg^–1^	–	117.60
Total Cd mg kg^–1^		0.61

*EC, electrical conductivity; N, nitrogen; K, potassium; P, phosphorous; C, carbon; Cd, cadmium.*

### Destructive Plant Sampling and Measurements

The maize plants were harvested at 70 DAS. The root and shoot lengths were determined using a measuring tape. Root and fresh shoot biomass were selected *via* an electronic weighing balance. At the same time, shoot and dry root biomass were determined after putting shoot/root samples in the oven at 75°C till constant weight (Virk et al., 2020). Leaf area was manually calculated by measuring leaf length, width, and multiplying with a correction factor of 0.75.

### Physiological Activities

Photosynthetic activity and chlorophyll content were recorded at 60 DAS. Chlorophyll content was measured using soil plant analysis development (SPAD) values of a fully expanded leaf with a SPAD meter (SPAD 502, Konica Minolta, Osaka, Japan). Crop photosynthetic activities including intercellular CO_2_ (Ci), transpiration rate (Tr), photosynthetic rate (Pn), and stomatal conductance (Gs) were measured with a portable IRGA analyzer (Infrared gas analyzer, Portable Gas Exchange Fluorescence System GFS-3000, Walz Heinz GmbH, Eichenring, Effeltrich, Germany). These data were collected between 10:00 and 12:00 h from a representative plant leaf.

### Oxidant and Antioxidant Activities

Activities of lipid peroxidation (MDA and H_2_O_2_) and electrolyte leakage (EL) were measured from fully developed leaves of the maize plant 60 DAS. EL of the maize leaves was measured according to the standard protocol. Prior to analysis, harvested leaves were washed with sterilized water to remove any trapped electrolyte and placed in glass tubes (with 10 ml of distilled water) and then incubated adequately for 6 h at 25°C on a shaker. Finally, the electrical conductivity (EC) was calculated (EC1). Subsequently, the samples were suitably placed for 2 h at 90°C and after reaching equilibrium at 25°C, the EC was determined (EC2) (Dionisio-Sese and Tobita, 1998). The percentage of EL was calculated using the following equation:


$$\text{EL}=\frac{\text{EC}\,1}{\text{EC}\,2}\times 100$$

The MDA and H_2_O_2_ activities were analyzed using the standard procedure. In brief, 0.1% thiobarbituric acid (TBA), with a volume of 5.0 ml, was added to 0.25 g leaves of maize, and the supernatant was centrifuged (12,000 r/min) at 4°C for 10 min. Then, 4 ml of 20% trichloroacetic acid (TCA) containing 0.5% was added to 1 ml of supernatant and heated at 95°C for 30 min. The mixture was cooled, and the mixture was again centrifuged (10,000 r/min) for 10 min under 4°C (Heath and Packer, 1968). The mixture was analyzed by absorbance at 450, 532, and 600 nm using a spectrophotometer (V-5800 visible spectrophotometer, Mesh Shanghai Yuanxi Instrument Co., Ltd., Shanghai, China). To determine H_2_O_2_, phosphate buffer solution (pH 6.5, 50 mM) at 3 ml was added in 50 mg maize leaf tissues and centrifuged (12,000 r/min) at 4°C for 30 min. Subsequently, 1 ml titanium sulfate in 20% sulfuric acid was gently mixed in the solution and again centrifuged (12,000 r/min) at 4°C for 20 min. Then, the absorption of the liquid was measured at 410 nm. The extermination coefficient of 0.28 μmol^–1^ cm^–1^ was managed to record H_2_O_2_ contents (Dionisio-Sese and Tobita, 1998).

Antioxidant activities, i.e., catalase (CAT), superoxide dismutase (SOD), and peroxidase (POD), were determined using a spectrophotometer (visible spectrophotometer V-5800, Mesh Shanghai Yuanxi Instrument Co., Ltd., Shanghai, China). Maize leaves were kept in liquid nitrogen. After standardization, maize leaves were ground with quartz sand (0.5 g), polyvinyl tyrosanone (PVP; 100 mg), and 0.05 M phosphate buffer (pH 7.8) in pestle and mortar. The supernatants were collected in centrifuged tubes and allowed to centrifuge (12,000 r/min) at 4°C for 10 min. CAT activity was determined according to Aebi (1984).

### Cadmium Determination in Maize Shoot, Root, and Soil

Dried shoot, root, and soil samples were ground correctly and allowed to sieve through a 0.5-mm sieve. The samples were digested in 10 ml of perchloric acid (HClO_4_) and nitric acid (HNO_3_) (1:3 v/v) overnight (Ryan et al., 2001). Subsequently, contents of Cd in the soil, shoot, and root were observed *via* an atomic absorption spectrophotometer (Model 3200-C, S/N: KETC0478, Company Heinz Walz GmbH, 91090, Effecltrich, Germany).

### Statistical Analyses

Collected data were subjected to ANOVA technique using SPSS statistical software (Version 25.0, SPSS Inc., Chicago, IL, United States). The average treatment means were compared using the least significant difference at *p* < 0.05. Graph Prism was used to create the graphical presentation. Pearson’s correlation analysis was performed to find a linear association between Cd concentration and maize plant physiology.

## Results

### Plant Morphology and Leaf Area

**FIGURE 1:**
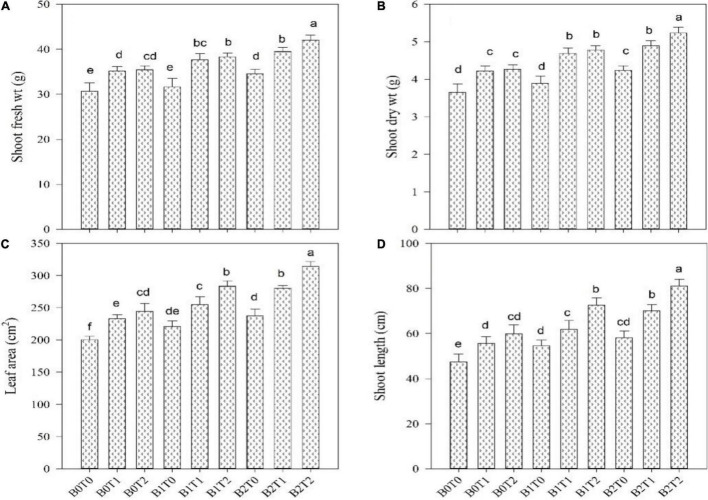
Effect of combined application of TU and BC on **(A)** shoot fresh weight, **(B)** shoot dry weight, **(C)** leaf area, and **(D)** shoot length grown under cadmium (Cd) contamination. Values are averages ± SD (*n* = 3). Different statistical letters represent significant differences in values between treatments (using Tukey’s multiple range test, *p* < 0.05). TU, thiourea; BC, biochar; *T*_0_ = 0 mg L^–1^ thiourea; *T*_1_ = 600 mg L^–1^ thiourea; *T*_2_ = 600 mg L^–1^ thiourea; *B*_0_ = 0% w/w BC; *B*_1_ = 2.5% w/w BC; *B*_2_ = 5.0% w/w BC.

**FIGURE 2:**
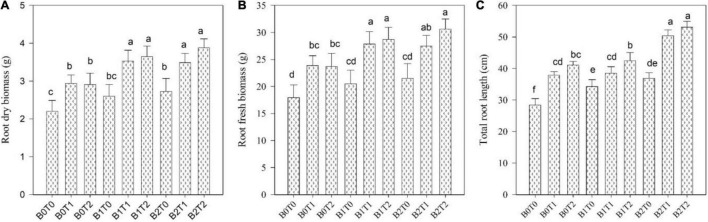
Effect of combined application of TU and BC on **(A)** root dry biomass, **(B)** root fresh biomass, and **(C)** total root length grown under Cd contamination. Values are averages ± SD (*n* = 3). Different statistical letters represent significant differences in values between treatments (*p* < 0.05). TU, thiourea; BC, biochar; *T*_0_ = 0 mg L^–1^; *T*_1_ = 600 mg L^–1^; *T*_2_ = 600 mg L^–1^; *B*_0_ = 0% w/w; *B*_1_ = 2.5% w/w; *B*_2_ = 5.0% w/w.

The root length, fresh root biomass, root dry biomass, shoot length, shoot fresh biomass, shoot dry biomass, and leaf area of maize were significantly altered by the application of BC and TU grown under Cd-contaminated soil (Figures 1, 2). Results revealed that contamination of Cd significantly reduced the root length, root fresh biomass, root dry biomass, shoot length, shoot fresh biomass, shoot dry biomass, and leaf area of maize (Figures 1, 2). However, incorporation of *B*_2_ in Cd-contaminated soil significantly improved the root length, fresh root biomass, root dry biomass, shoot length, shoot fresh biomass, shoot dry biomass, and leaf area by 23, 17, 19, 18, 11, 14, and 15%, respectively, more outstanding in comparison with control. Similarly, alone foliar application of TU (*T*_2_), enhanced the root length, fresh root biomass, root dry biomass, shoot length, shoot fresh biomass, shoot dry biomass, and leaf area by 31, 24, 24, 21, 13, 14, and 18%, respectively, more outstanding in comparison with control. Correspondingly, combined application of *T*_2_ and *B*_2_ tends to improve the root length, fresh root biomass, root dry biomass, shoot length, shoot fresh biomass, shoot dry biomass, and leaf area as they were 47, 41, 43, 42, 27, 30, and 36%, respectively, higher as compared with control.

### Oxidant and Antioxidant Activity

**FIGURE 3:**
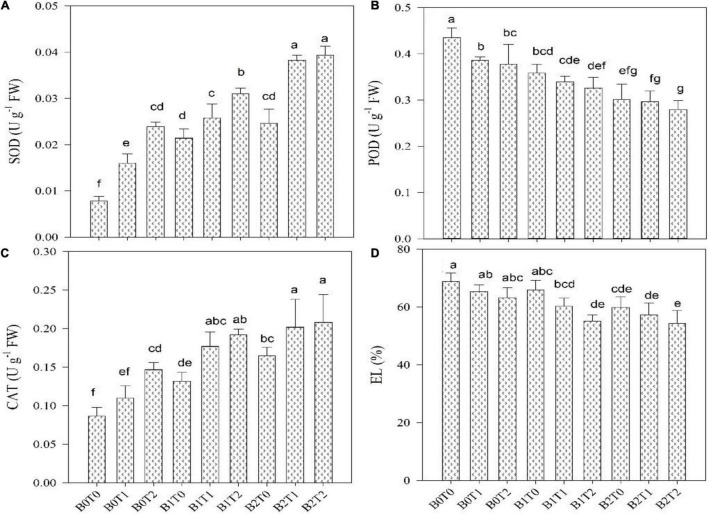
Effect of combined application of TU and BC on **(A)** superoxide dismutase (SOD), **(B)** peroxidase (POD), **(C)** catalase (CAT), and **(D)** electrolyte leakage (EL), grown under Cd contamination. Values are averages ± SD (*n* = 3). Different statistical letters represent significant differences in values between treatments (*p* < 0.05). TU, thiourea; BC, biochar; *T*_0_ = 0 mg L^–1^; *T*_1_ = 600 mg L^–1^; *T*_2_ = 600 mg L^–1;^ *B*_0_ = 0% w/w; *B*_1_ = 2.5% w/w; *B*_2_ = 5.0% w/w.

**FIGURE 4:**
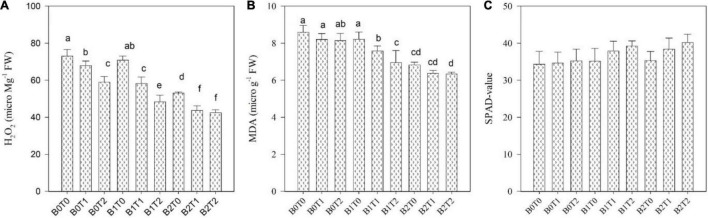
Effect of combined application of TU and BC on **(A)** hydrogen peroxide (H_2_O_2_), **(B)** malondialdehyde (MDA), and **(C)** SPAD value grown under Cd contamination. Values are averages ± SD (*n* = 3). Different statistical letters represent significant differences in values between treatments (*p* < 0.05). TU, thiourea; BC, biochar; *T*_0_ = 0 mg L^–1^; *T*_1_ = 600 mg L^–1^; *T*_2_ = 600 mg L^–1;^ *B*_0_ = 0% w/w; *B*_1_ = 2.5% w/w; *B*_2_ = 5.0% w/w.

Oxidative stress values, i.e., MDA, H_2_O_2_, and EL, were significantly enhanced in maize leaves under Cd stress (Figures 3, 4). Highest MDA, H_2_O_2_, and EL values in maize leaves were recorded in control (*B*_0_*T*_0_), i.e., with the mean values of 9, 153, and 69%, respectively. However, results revealed that incorporating *B*_2_ in soil minimizes the MDA, H_2_O_2_, and EL values in maize leaves by 20, 13, and 13%, respectively, compared with control. Likewise, exogenous application of *T*_2_ significantly minimized the MDA, H_2_O_2_, and EL values by 5, 9, and 8%, respectively, compared with control. Correspondingly, combined *T*_2_ and *B*_2_ reduced the MDA, H_2_O_2_, and EL values by 26, 20, and 21%, respectively, lower than *B*_0_*T*_0_. The antioxidant activities, i.e., SOD, CAT, and POD in maize leaves, were significantly altered under both TU and BC addition under Cd stress (Figures 3, 4). The highest POD activity was recorded in the control treatment (*B*_0_*T*_0_), having a mean value of 0.44 μ g^–1^ FW. The combined exogenous application of *T*_2_ and *B*_2_ decreases the POD activity in maize leaves by 36% than that of the control. Likewise, SOD and CAT activities were significantly improved in maize leaves by exogenous foliar application of TU and BC incorporation in soil. The highest SOD (0.040 μ g^–1^ FW) and CAT (0.21 μ g^–1^ FW) activities were in the combined exogenous foliar application of BC and TU (*B*_2_*T*_2_), which were 81 and 59% higher in comparison with *B*_0_ (0.0074 μ g^–1^ FW) and *T*_0_ (0.087 μ g^–1^ FW).

### Photosynthesis Activity, Soil Plant Analysis Development Value, and Gas-Exchange Parameters

**FIGURE 5:**
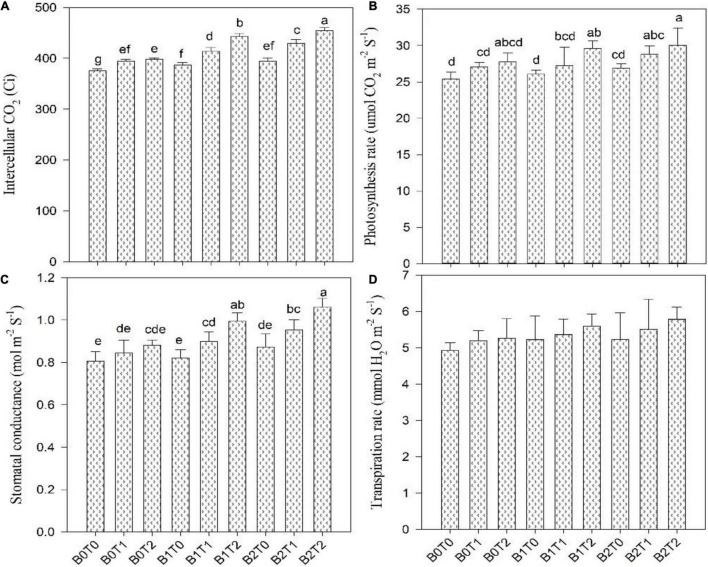
Effect of combined application of TU and BC on **(A)** intercellular CO_2_, **(B)** photosynthesis rate, **(C)** stomatal conductance, and **(D)** transpiration rate grown under Cd contamination. Values are averages ± SD (*n* = 3). Different statistical letters represent significant differences in values between treatments (*p* < 0.05). TU, thiourea; BC, biochar; *T*_0_ = 0 mg L^–1^; *T*_1_ = 600 mg L^–1^; *T*_2_ = 600 mg L^–1;^ *B*_0_ = 0% w/w; *B*_1_ = 2.5% w/w; *B*_2_ = 5.0% w/w.

Photosynthesis activity and gas-exchange parameters, i.e., stomatal conductance and intercellular CO_2_ concentration, were significantly affected in maize leaves under Cd stress; however, the SPAD value and transpiration rate results were non-significant under Cd stress (Figures 4, 5). Results revealed that contamination of Cd significantly reduced the photosynthesis activity, SPAD value, and gas-exchange parameters of maize. However, incorporating *B*_2_ in Cd-contaminated soil significantly improved photosynthesis activity, SPAD value, and gas-exchange parameters of maize. Concerning BC, the highest photosynthesis activity, SPAD value, stomatal conductance, transpiration rate, and intercellular CO_2_ concentration were recorded in *B*_2_ that were 6, 3, 8, 6, and 5% higher in comparison with control (*B*_0_*T*_0_), respectively. Likewise, exogenous application of TU_2_ enhanced the photosynthesis activity, SPAD value, stomatal conductance, transpiration rate, and intercellular CO_2_ concentration by 9, 3, 9, 7, and 6%, respectively, higher as compared with control. The most increased photosynthesis activity, SPAD value, stomatal conductance, transpiration rate, and intercellular CO_2_ concentration were recorded in the combined application of *B*_2_*T*_2_ that were 15, 15, 25, 15, and 17%, respectively, higher in comparison with control.

### Cadmium Concentration in Different Plant Parts

**FIGURE 6:**
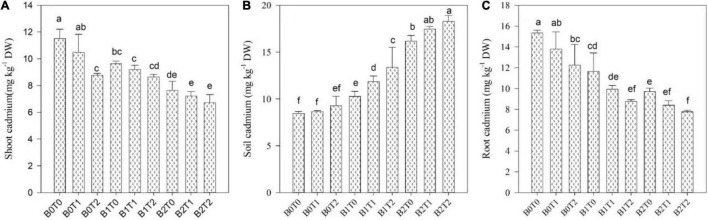
Effect of combined application of TU and BC on **(A)** Cd concentration in shoot, **(B)** Cd concentration in soil, and **(C)** Cd concentration in root grown under Cd contamination. Values are averages ± SD (*n* = 3). Different statistical letters represent significant differences in values between treatments (*p* < 0.05). TU, thiourea; BC, biochar; *T*_0_ = 0 mg L^–1^; *T*_1_ = 600 mg L^–1^; *T*_2_ = 600 mg L^–1;^ *B*_0_ = 0% w/w; *B*_1_ = 2.5% w/w; *B*_2_ = 5.0% w/w.

Cadmium accumulation in soil and various maize components, i.e., shoot and root, significantly impacts the two-way association between BC and TU (Figure 6). With all forms of Cd contamination, the Cd concentration in soil was usually the highest relative to the Cd concentration in maize shoot and root. The plant-available Cd in maize shoot and root was significantly reduced after incorporating BC into the soil. The incorporation of BC reduced the Cd concentration in the shoot and root of maize plants by 33 and 37%, respectively, compared with *B*_0_*T*_0_. Likewise, exogenous application of TU minimized the Cd concentration in shoot and root of maize plants by 24 and 20%, respectively, than that of *B*_0_*T*_0_. Correspondingly, the combined application of *B*_2_*T*_2_ minimized the Cd concentration in shoot and root of maize plants by 42 and 49% compared with *B*_0_*T*_0_, respectively.

### Correlation Analysis and Principal Component Analysis

**FIGURE 7:**
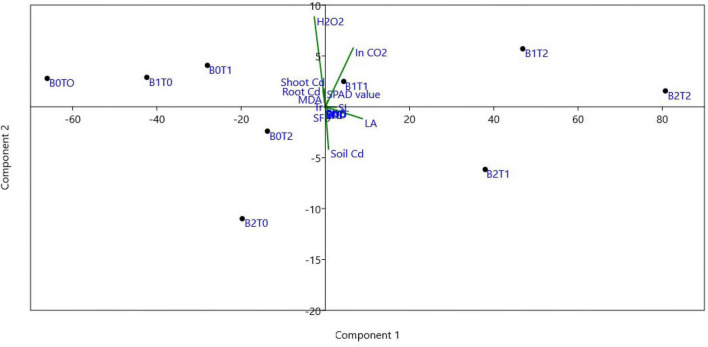
Principal component analysis (PCA) of all parameters for treatment combination of TU and BC. *T*_0_ = 0 mg L^–1^; *T*_1_ = 600 mg L^–1^; *T*_2_ = 600 mg L^–1^; *B*_0_ = 0% w/w; *B*_1_ = 2.5% w/w; *B*_2_ = 5.0% w/w. BC, biochar; TU, thiourea; RL, total root length; RFB, root fresh biomass; RDB, root dry biomass; SL, shoot length; SFB, shoot fresh biomass; SDB, shoot dry biomass; LAI, leaf area index; SOD, superoxide dismutase in leaves; POD, peroxidase in leaves; CAT, catalase in leaves; EL, electrolyte leakage in leaves; MDA, malondialdehyde; H_2_O_2_, hydrogen peroxide; Ci, intercellular CO_2_ concentration; Pn, net photosynthesis rate; Gs, stomatal conductance; Tr, transpiration rate; SPAD, SPAD value; Soil Cd, cadmium concentration in soil; Shoot Cd, cadmium concentration in maize shoot; Root Cd, cadmium concentration in root.

Correlation analysis showed that root indicators are strongly interrelated with the growth parameters such as shoot length, shoot dry biomass, and leaf area index (Table 2). Most of the determined enzymatic activities, i.e., SOD, POD, EL, MDA, and H_2_O_2_, were negatively (*p* < 0.05) correlated with the maize growth indicators. In contrast, the values of CAT were positively correlated with maize growth parameters. Similarly, the correlation between stomatal conductance and POD (-0.71), EL (0.81), MDA (-0.69), and H_2_O_2_ (-0.78) were negatively linked at *p* < 0.05 statistical significance. Specifically, Cd concentration adversely affected (*p* < 0.05) enzymatic activities of maize plants, which eventually decreased plant growth. The principal component analysis (PCA) demonstrated that the combined application of BC and TU decreased plant Cd and increased soil Cd (Figure 7).

**TABLE 2:**
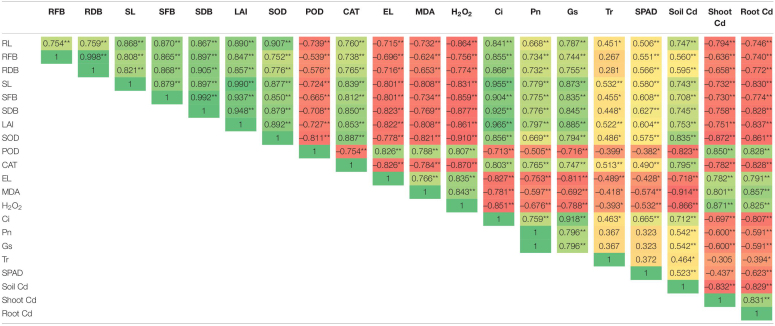
Pearson’s association between .various traits in maize (*n* = 3) grown under Cd contamination

**represents that results are significant at p < 0.01; *represents that results are significant at p < 0.05; RL, total root length; RFB, root fresh biomass; RDB, root dry biomass; SL, shoot length; SFB, shoot fresh biomass; SDB, shoot dry biomass; LAI, leaf area index; SOD, superoxide dismutase in leaves; POD, peroxidase in leaves; CAT, catalase in leaves; EL, electrolyte leakage in leaves; MDA, malondialdehyde; H_2_O_2_, hydrogen peroxide; Ci, intercellular CO_2_ concentration; Pn, net photosynthesis rate; Gs, stomatal conductance; Tr, transpiration rate; SPAD, SPAD value; Soil Cd, cadmium concentration in soil; Shoot Cd, cadmium concentration in maize shoot; Root Cd, cadmium concentration in root.

## Discussion

The plant growth is affected when agricultural land is contaminated with high levels of Cd. To reduce Cd toxicity in plants, it is essential to mitigate Cd uptake and accumulation. Maize plants exposed to Cd toxicity showed impaired growth (Tanveer and Shabala, 2022). Also, in Cd-toxicity situations, however, the incorporation of BC and TU alone or in combined form recovered these declines in growth attributes (Figures 2, 7 and Table 2). The addition of BC and TU in the combined form was found to be the most effective in reducing the adverse effects of Cd. This should be clarified in terms of their roles. Exogenous application of TU protects the degradation of chlorophyll contents under Cd stress. In addition, TU significantly minimized the Cd concentrations in various plant tissues of maize (Figure 6), which may be attributed to the function of TU in cell wall lignifications. Cd stress lowers leaf chlorophyll concentrations and causes oxidative stress to photosynthetically active cell development (Figure 5). The replacement of magnesium ions with Cd ions in chlorophyll molecules causes a decrease in chlorophyll contents since the proportion conversion of chlorophyll to pheophytin enhances with increased Cd stress. Carotenoids are known to protect chlorophyll and other essential macromolecules from free radical production by lowering the excited triplet state of chlorophyll (Dad et al., 2021). In this study, the synergistic effect of TU and BC induced maize growth enhancement due to greater chlorophyll concentrations and photosynthesis activity under Cd stress.

Lignification is a process comprised of lignin formation, where complex phenolic-heteropolymers are found in cell walls. In plants, these phenolic metabolites may connect to the plant cell wall, reducing the accumulation and assimilation of trace-metal contaminates in plant tissues (Khanna et al., 2019b). Moreover, root lignification can limit the Cd-apoplastic pathway and its translocation from the root zone to the plant aboveground part, i.e., stem, flowers, and leaves (Khanna et al., 2019c). Lignification is a multienzymatic mechanism mediated by various exogenous plant hormones in plants. TU contributes to regulating metabolic processes, including the phenolic pathways, during the lignification process (Perveen et al., 2015). BC is enriched with cations, i.e., Mg^+2^, K^+^, and Ca^+2^. These cation ions might be embedded in the cell walls of plant tissues such as amorphous buffers, reducing Cd absorption, and uptake from the rhizosphere to plants (Younis et al., 2016).

Furthermore, plants can competitively absorb cations, Cd, Mg^+2^, K^+^, and Ca^+2^ compete with Cd for exchange sites in soils, and Cd absorption in soils can be decreased by the presence of competitive ions, i.e., Mg^+2^, K^+^, and Ca^+2^. BC and TU had a synergistic effect, indicating that the two together were more effective at reducing the Cd toxicity than either one alone. Under Cd stress, maize treated with both BC and TU had significantly higher plant height, above, and below-ground biomass than maize treated with the Cd only (Xu et al., 2014).

Appropriate exogenous application of soil amendment or plant growth hormones reduces the health hazards and metal contamination in an environmentally sustainable manner (Chaoui and El Ferjani, 2005). The porous structure of BC improves the water holding capacity of the plant and maintains the photosynthesis activity of the plant under Cd stress. In addition, BC may also increase soil conditions by improving the plant absorption of macronutrients/micronutrients (Mg, Ca, P, K, Mn, Fe, and Cu) (Retamal-Salgado et al., 2017), rendering it an excellent agricultural fertilizer source. Increasing the content of macronutrients in plant tissues, TU can also increase the nutrient content in soil (Ge et al., 2019). These findings suggest that the combination of BC and TU could significantly improve the nutrient content and thereby reduce Cd uptake in root and shoot parts of maize. Nutrients essential for plants can affect Cd uptake, aggregation, translocation, bioavailability, and dispersal by adsorbing Cd on the surface, chelating or binding with Cd, thereby reducing Cd assimilation, uptake, and availability. Therefore, the improvement in maize growth observed in this research following the addition of BC and TU showed the combined effect of BC and TU in reducing Cd accumulation in maize. This research illustrates that exogenous application of BC and TU has a combined remediation effect on Cd-induced stress, implying that BC as a soil improver in conjunction with foliar application of maize with TU may be a potentially useful way to reduce Cd toxicity in agricultural development.

Under normal development circumstances, plants usually maintain a balance between ROS formation and elimination; however, this equilibrium is disrupted by Cd stress conditions. ROS release under a stressful situation causes lipid peroxidation, which contributes to cell membrane permeability, as demonstrated in this study. Cell membrane permeability is enhanced in response to Cd stress (Figures 3, 4). The plasma membrane may be damaged by various mechanisms, including the oxidation reaction caused by ROS (Fahad et al., 2016). It was reported that Cd has a significant attraction to nitrogen and sulfur-containing amino acids, ligands, and proteins. As a result, it binds to proteins, disrupting membrane ion channels and causing ion leakage (Dad et al., 2021). In contrast, the BC and TU application mitigated the harmful effects of Cd impact on lipid peroxidation and cell membrane permeability. Under Cd stress, BC and TU application decreased EL and MDA contents by 69 and 9%, respectively, compared with Cd stress alone (Figures 3D, 4B), suggesting that TU-BC might enhance Cd tolerance in maize by retaining cell membrane permeability. BC may reduce oxidative stress by mitigating ROS generation and improving antioxidant activity (Fahad et al., 2016). When antioxidant activity rises in response to BC, it aids in the prevention of the start or expansion of oxidizing chain reactions, resulting in inhabitation or oxidation delays in plant cells, lipids, proteins, and other essential components (Abbas et al., 2017; Liu et al., 2018). Excessive ROS generation is regulated by antioxidant activation as an adaptive response; enhanced antioxidant activity does not imply better ROS detoxification. The activities of SOD and CAT were modulated in response to TU and BC application under Cd stress in the current investigation. TU and BC application increases CAT and SOD activities (Figures 3A,C), which was negatively connected with MDA levels and positively correlated with the improved shoot and root biomass accumulation under Cd stress (Figure 4B).

The foliar spray of TU causes Cd to accumulate in the root cell walls in rice, which is analogous to the positive effect of BC under Cd-stress agricultural soil. The contribution of plant cell walls in resistance against trace metal toxicity is imperative (Ahmad et al., 2016). As the first line of defense against toxic effects, the cell wall supplies a critical aptitude for Cd storage and accumulation in plants. It reacts effectively and rapidly to trace-metal contamination. The absorption of Cd in plants can be inhibited by the ability of the cell wall to compare Cd (Zhang et al., 2019; Wang et al., 2020). The critical components of the plant cell wall are matrix polysaccharides and cellulose. Polygalacturonic acids bind the majority of trace metals found in cell walls. In plants, cell walls may give functional groups, i.e., pectin, cellulose-microfibrils, and lignin, to unite Cd ions simultaneously and limit their bioavailability (Khanna et al., 2019a; Feng et al., 2020). TU can reduce Cd toxicity in cereals by enhancing hemicellulose and pectin content in root cell walls, improving the accumulation of Cd in cell walls of the root zone, and decreasing the accumulation of Cd in the below-aboveground fraction (Zhang et al., 2009). Cd translocation from the root zone to shoots is thought to be a key mechanism for increasing plant Cd tolerance. Our findings suggest that BC and TU are active in the deposition of Cd in cell walls or maybe the Cd-chelation reaction in maize plants, reducing Cd toxicity (Figures 6, 7). Furthermore, the combined utilization of BC and TU could have the same improving behavior as other trace metals.

The use of BC and TU reduces the Cd content in plant tissues by minimizing the bioaccumulation of the metal in the plant and its translocation from the soil to the plant. Enrichment of BC can reduce the Cd content in the biosphere by increasing soil pH and by various mechanisms, i.e., complexation, physical sorption, precipitation, membrane filtration, ion exchange, and electrostatic interactions, thereby affecting metal speciation in the rhizosphere and reducing Cd availability (Sfaxi-Bousbih et al., 2010; Ali et al., 2013). Changes in Cd adsorption caused by BC and TU arbitrated variations in the nutrient contents in the soil can reduce the uptake of Cd by maize and its subsequent accumulation to stem and grain of maize (Ahmad et al., 2018). Likewise, exogenous foliar application of TU has been shown to improve rice Cd resistance by increasing hemicelluloses and pectin contents in the root cell wall (Perveen et al., 2015). BC also comprises micronutrients such as silicon (Si), and the wall-bound Si in the rice cell can inhibit the Cd uptake. Decreased Cd accumulation in maize, shoot, and root parts under combined application of *B*_2_*T*_2_ can be attributed to a non-availability and lower Cd uptake. Exogenous addition of BC or TU to cereal plants, i.e., wheat, rice, sorghum, maize, and barley, could alter the Cd distribution. In recent years, it was reported that BC incorporation suppresses the gene expression involved in Cd transport and Cd uptake, as well as apoplastic Cd translocation to shoots, potentially reducing Cd toxicity. Due to its high mobility and solubility, plants grown in Cd-contaminated soils can consume Cd easily (Abbas et al., 2017). Cd absorption causes photosynthesis inhibition, chlorophyll depletion, growth retardation, and most significantly, accumulation of Cd in maize edible parts such as grain, resulting in adverse and even lethal effects for the health of human beings *via* contaminating the food chain (Kamnev and Van Der Lelie, 2000). So, combining the application of BC and TU to maize could increase the maize growth and grain quality while also lowering the human health risk allied with consuming Cd-contaminated food. The utilization of BC and TU in combination could offer a cost-effective and environmentally safe way to reduce Cd toxicity. Furthermore, BC fertilizers are now inexpensive, and the quantity of TU added is lower, all of which are practical for practical applications.

## Conclusion

Early identification of Cd translocation and accumulation is critical to deal with food-safety issues, and this necessitates the in-season measurement of Cd accumulation in plants. Overall, the experiment results verify our hypothesis that combined application of BC and TU decrease Cd concentration in plant organs and improve maize growth and development. Specifically, a higher application rate of *B*_2_ (5% w/w) and *T*_2_ (1,200 mg L^–1^) significantly decreases Cd concentration in maize shoot and root. However, higher soil Cd was present under *B*_2_*T*_2_ due to lower plant uptake than *B*_0_*T*_0_. A higher application rate of TU and BC also improved crop growth by increasing root dry biomass, root fresh biomass, and total root length as compared with *B*_0_*T*_0_. The maize physiological activities, such as intercellular CO_2_, photosynthetic rate, and stomatal conductance, were significantly improved under *B*_2_*T*_2_ and *B*_2_*T*_1_ treatments. Overall, our results suggest that the combined application of a higher level of *B*_2_*T*_2_ (*B*_2_; 5% w/w, *T*_2_; 1,200 mg L^–1^) not only provides a simple and enumerate agronomic management strategy for reducing Cd concentration in maize plant organs but also improves its physiological activities in Cd contaminated soil. Nevertheless, further farming studies should be seriously investigated in the future to conclude a more precise and effective strategy for reducing Cd bioavailability in the rhizosphere.

## Data Availability Statement

The original contributions presented in the study are included in the article/Supplementary Material, further inquiries can be directed to the corresponding authors.

## Author Contributions

FU, CL, AL, and MR: conceptualization. FU and AL: methodology. FU, AL, and MR: validation and formal analysis. NA, AL, and AS: investigation and data curation. FU, AL, MR, SA-U-K, CL, WS, MS, MB, and AS: writing—original draft preparation, review, and editing. CL, AL, and MR: supervision and project administration. All authors contributed to the article, read, and agreed to the published version of the manuscript.

## Conflict of Interest

The authors declare that the research was conducted in the absence of any commercial or financial relationships that could be construed as a potential conflict of interest.

## Publisher’s Note

All claims expressed in this article are solely those of the authors and do not necessarily represent those of their affiliated organizations, or those of the publisher, the editors and the reviewers. Any product that may be evaluated in this article, or claim that may be made by its manufacturer, is not guaranteed or endorsed by the publisher.
