# Supplementary material for: Integrated Application of Thiourea and Biochar Improves Maize Growth, Antioxidant Activity and Reduces Cadmium Bioavailability in Cadmium-Contaminated Soil
Source: Front Plant Sci. 2022 Jan 28;12:809322. doi: 10.3389/fpls.2021.809322 (PMC8845445; doi:10.3389/fpls.2021.809322)
Supplement: Supplementary file 1 [file Table_1.docx]

Table S1: Weather conditions during experimental period, Lanzhou, Gansu, China

|  | **Months** | | |
| --- | --- | --- | --- |
| **Weather parameters** | July | August | September |
| Minimum temperature °C | 17 °C | 16 °C | 11.2 °C |
| Maximum temperature °C | 28.7 °C | 26.9 °C | 21.2 °C |
| Humidity % | 50% | 55% | 63% |
| Sunshine duration (hours) | 10.9 | 9.4 | 7.4 |
| Rainfall mm (in) | 90 (3.5) | 104 (4.1) | 76 (3) |
